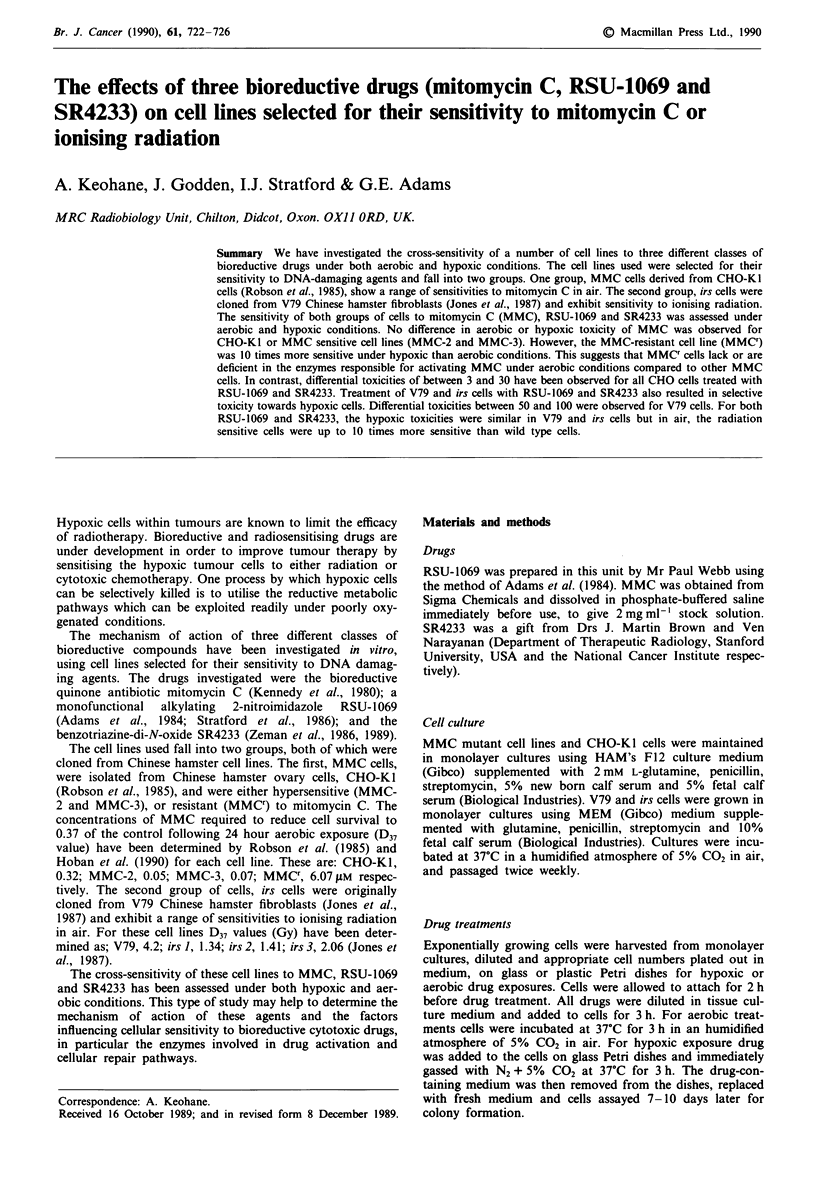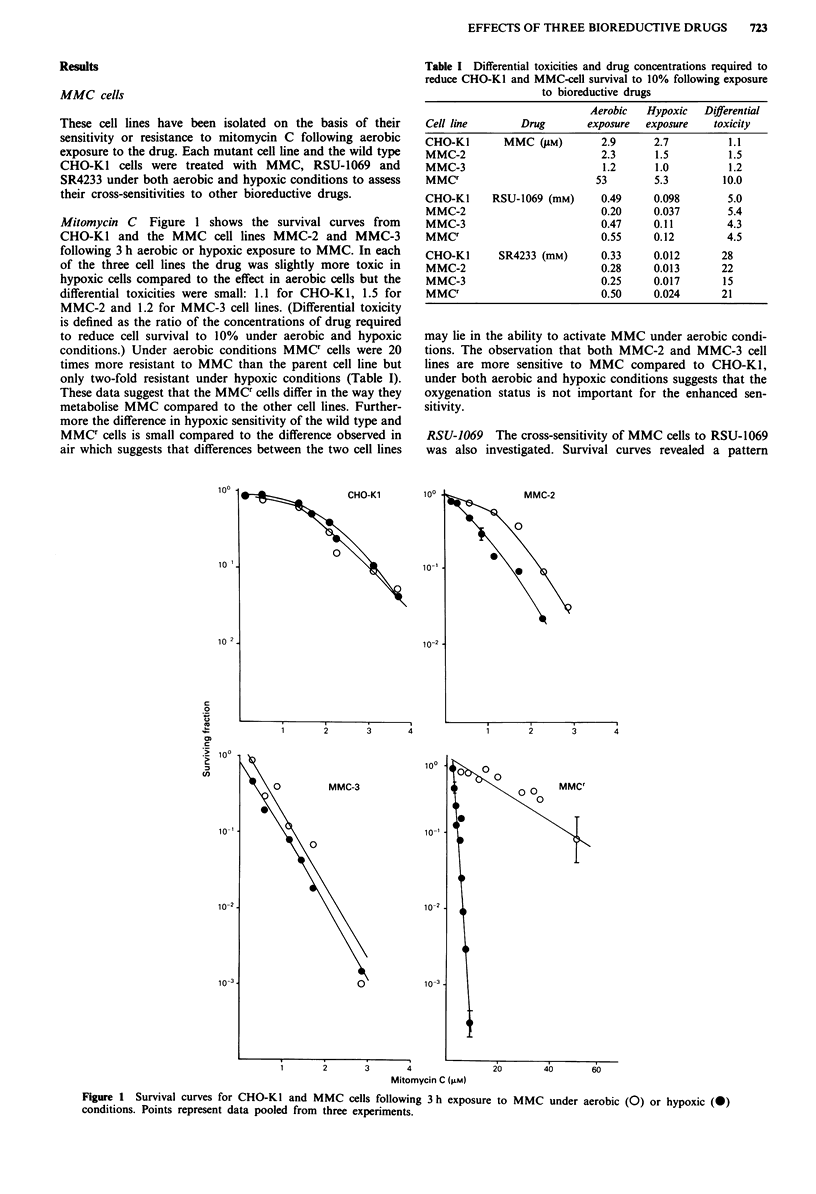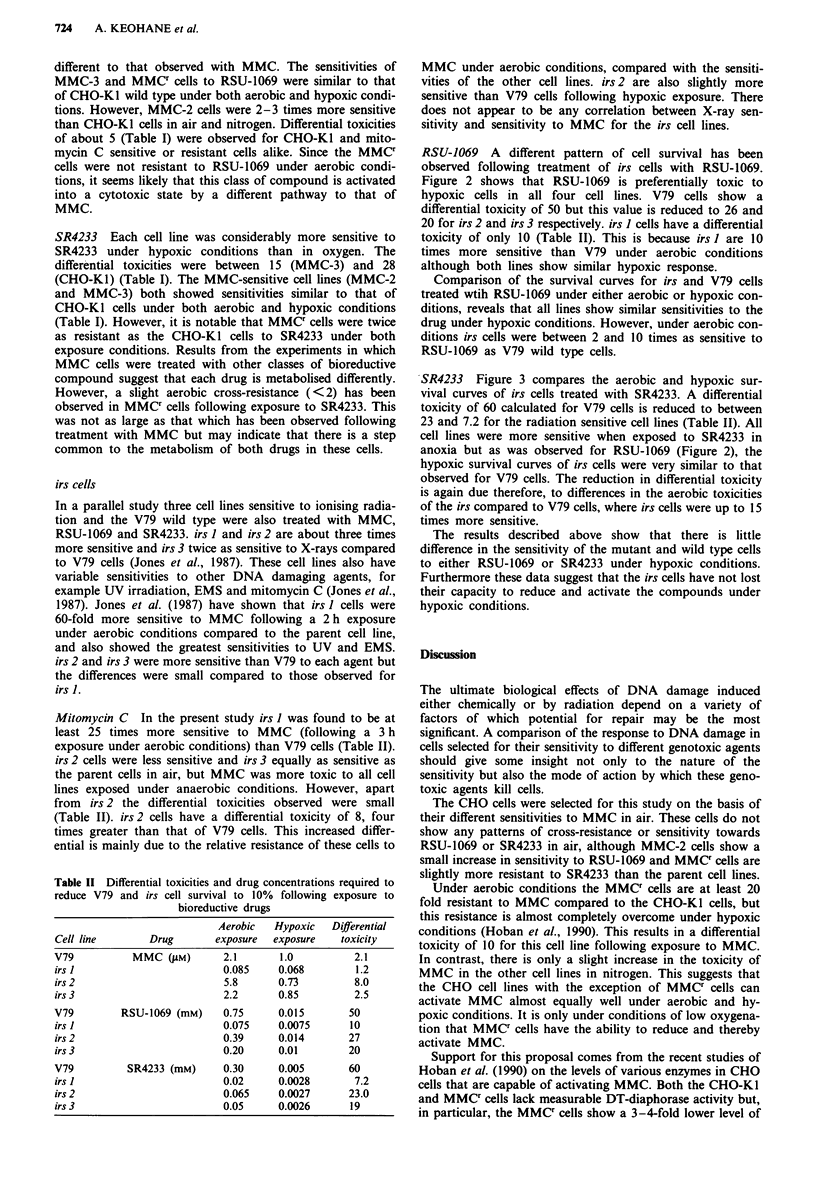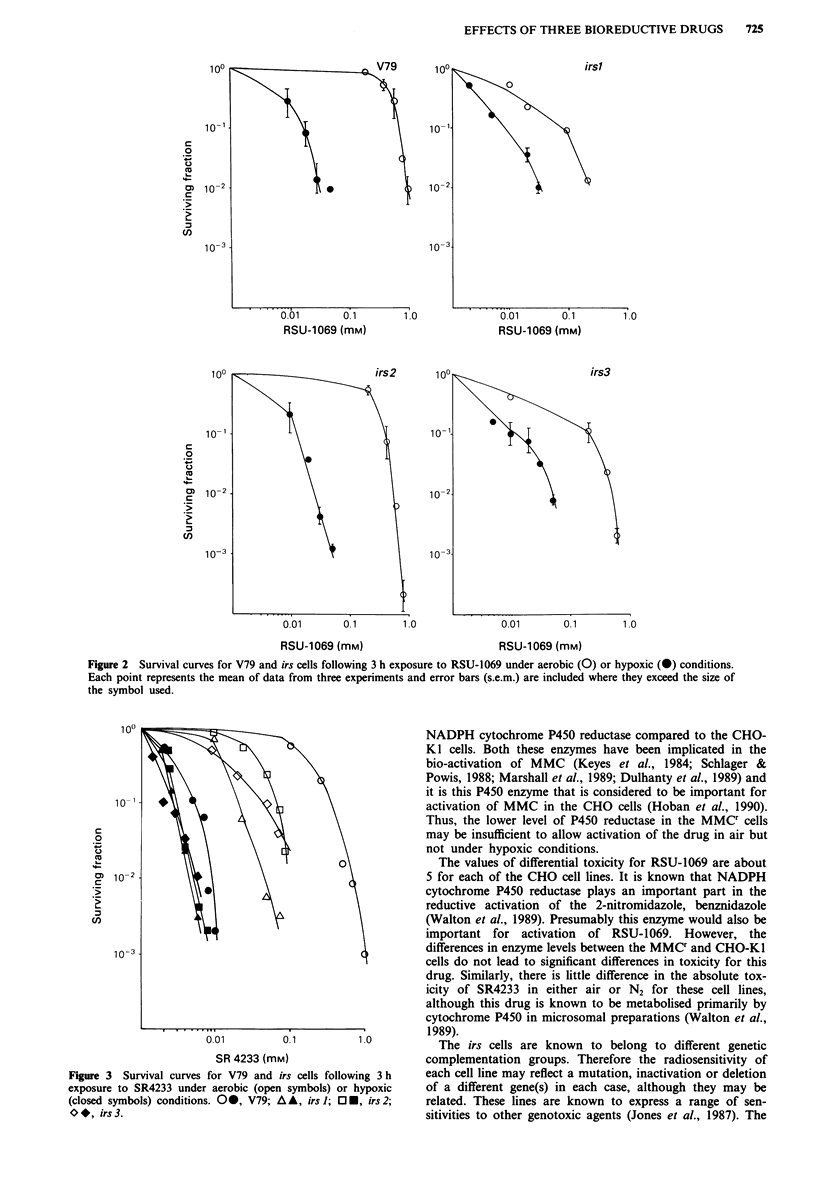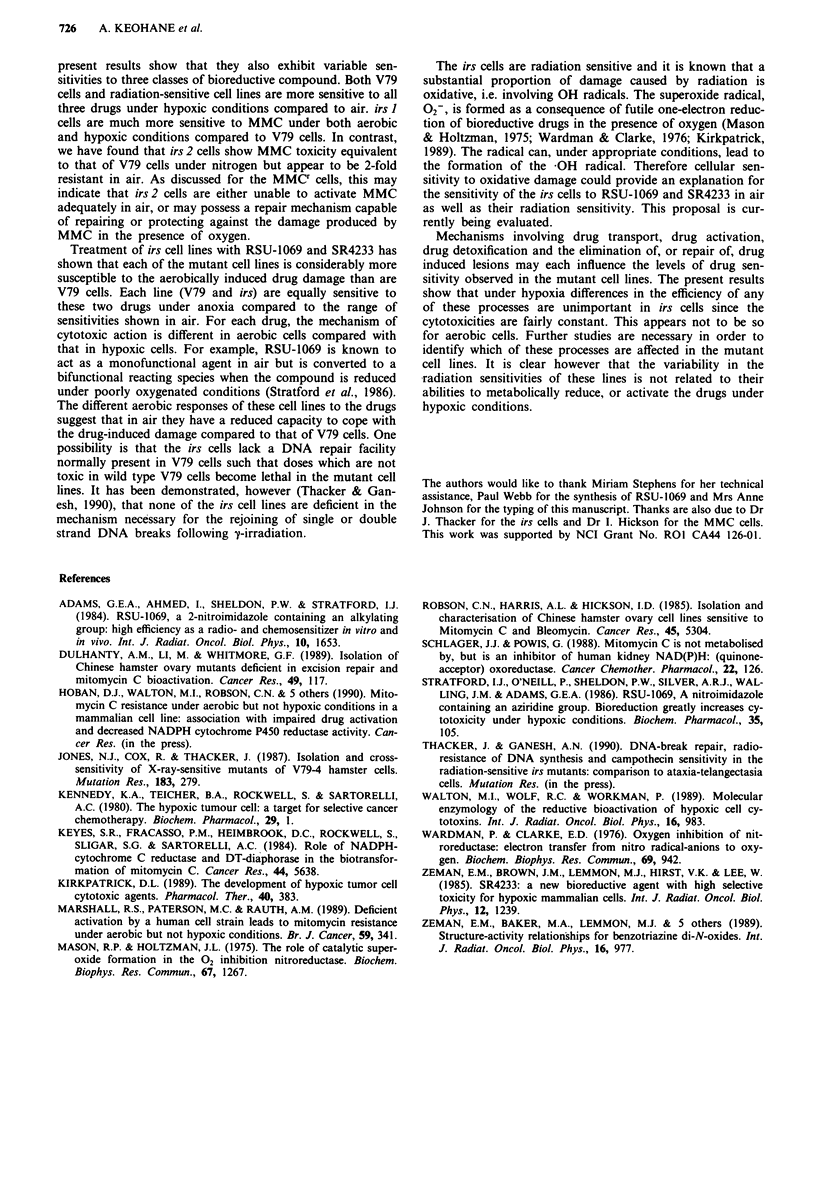# The effects of three bioreductive drugs (mitomycin C, RSU-1069 and SR4233) on cell lines selected for their sensitivity to mitomycin C or ionising radiation.

**DOI:** 10.1038/bjc.1990.162

**Published:** 1990-05

**Authors:** A. Keohane, J. Godden, I. J. Stratford, G. E. Adams

**Affiliations:** MRC Radiobiology Unit, Chilton, Didcot, Oxon, UK.

## Abstract

We have investigated the cross-sensitivity of a number of cell lines to three different classes of bioreductive drugs under both aerobic and hypoxic conditions. The cell lines used were selected for their sensitivity to DNA-damaging agents and fall into two groups. One group, MMC cells derived from CHO-K1 cells (Robson et al., 1985), show a range of sensitivities to mitomycin C in air. The second group, irs cells were cloned from V79 Chinese hamster fibroblasts (Jones et al., 1987) and exhibit sensitivity to ionising radiation. The sensitivity of both groups of cells to mitomycin C (MMC), RSU-1069 and SR4233 was assessed under aerobic and hypoxic conditions. No difference in aerobic or hypoxic toxicity of MMC was observed for CHO-K1 or MMC sensitive cell lines (MMC-2 and MMC-3). However, the MMC-resistant cell line (MMCr) was 10 times more sensitive under hypoxic than aerobic conditions. This suggests that MMCr cells lack or are deficient in the enzymes responsible for activating MMC under aerobic conditions compared to other MMC cells. In contrast, differential toxicities of between 3 and 30 have been observed for all CHO cells treated with RSU-1069 and SR4233. Treatment of V79 and irs cells with RSU-1069 and SR4233 also resulted in selective toxicity towards hypoxic cells. Differential toxicities between 50 and 100 were observed for V79 cells. For both RSU-1069 and SR4233, the hypoxic toxicities were similar in V79 and irs cells but in air, the radiation sensitive cells were up to 10 times more sensitive than wild type cells.


Br. J. Cancer (1990), 61, 722-726 © Macmillan Press Ltd., 1990

### The effects of three bioreductive drugs (mitomycin C, RSU-1069 and SR4233) on cell lines selected for their sensitivity to mitomycin C or ionising radiation

A. Keohane, J. Godden, I.J. Stratford & G.E. Adams

*MRC Radiobiology Unit, Chilton, Didcot, Oxon. OX11 0RD, UK.*

**Summary** We have investigated the cross-sensitivity of a number of cell lines to three different classes of bioreductive drugs under both aerobic and hypoxic conditions. The cell lines used were selected for their sensitivity to DNA-damaging agents and fall into two groups. One group, MMC cells derived from CHO-K1 cells (Robson *et al.*, 1985), show a range of sensitivities to mitomycin C in air. The second group, *irs* cells were cloned from V79 Chinese hamster fibroblasts (Jones *et al.*, 1987) and exhibit sensitivity to ionising radiation. The sensitivity of both groups of cells to mitomycin C (MMC), RSU-1069 and SR4233 was assessed under aerobic and hypoxic conditions. No difference in aerobic or hypoxic toxicity of MMC was observed for CHO-K1 or MMC sensitive cell lines (MMC-2 and MMC-3). However, the MMC-resistant cell line ($MMC^r$) was 10 times more sensitive under hypoxic than aerobic conditions. This suggests that $MMC^r$ cells lack or are deficient in the enzymes responsible for activating MMC under aerobic conditions compared to other MMC cells. In contrast, differential toxicities of between 3 and 30 have been observed for all CHO cells treated with RSU-1069 and SR4233. Treatment of V79 and *irs* cells with RSU-1069 and SR4233 also resulted in selective toxicity towards hypoxic cells. Differential toxicities between 50 and 100 were observed for V79 cells. For both RSU-1069 and SR4233, the hypoxic toxicities were similar in V79 and *irs* cells but in air, the radiation sensitive cells were up to 10 times more sensitive than wild type cells.

Hypoxic cells within tumours are known to limit the efficacy of radiotherapy. Bioreductive and radiosensitising drugs are under development in order to improve tumour therapy by sensitising the hypoxic tumour cells to either radiation or cytotoxic chemotherapy. One process by which hypoxic cells can be selectively killed is to utilise the reductive metabolic pathways which can be exploited readily under poorly oxygenated conditions.

The mechanism of action of three different classes of bioreductive compounds have been investigated *in vitro*, using cell lines selected for their sensitivity to DNA damaging agents. The drugs investigated were the bioreductive quinone antibiotic mitomycin C (Kennedy *et al.*, 1980); a monofunctional alkylating 2-nitroimidazole RSU-1069 (Adams *et al.*, 1984; Stratford *et al.*, 1986); and the benzotriazine-di-*N*-oxide SR4233 (Zeman *et al.*, 1986, 1989).

The cell lines used fall into two groups, both of which were cloned from Chinese hamster cell lines. The first, MMC cells, were isolated from Chinese hamster ovary cells, CHO-K1 (Robson *et al.*, 1985), and were either hypersensitive (MMC-2 and MMC-3), or resistant ($MMC^r$) to mitomycin C. The concentrations of MMC required to reduce cell survival to 0.37 of the control following 24 hour aerobic exposure ($D_{37}$ value) have been determined by Robson *et al.* (1985) and Hoban *et al.* (1990) for each cell line. These are: CHO-K1, 0.32; MMC-2, 0.05; MMC-3, 0.07; $MMC^r$, 6.07 μM respectively. The second group of cells, *irs* cells were originally cloned from V79 Chinese hamster fibroblasts (Jones *et al.*, 1987) and exhibit a range of sensitivities to ionising radiation in air. For these cell lines $D_{37}$ values (Gy) have been determined as; V79, 4.2; *irs 1*, 1.34; *irs 2*, 1.41; *irs 3*, 2.06 (Jones *et al.*, 1987).

The cross-sensitivity of these cell lines to MMC, RSU-1069 and SR4233 has been assessed under both hypoxic and aerobic conditions. This type of study may help to determine the mechanism of action of these agents and the factors influencing cellular sensitivity to bioreductive cytotoxic drugs, in particular the enzymes involved in drug activation and cellular repair pathways.

Correspondence: A. Keohane.
Received 16 October 1989; and in revised form 8 December 1989.

### Materials and methods

### Drugs

RSU-1069 was prepared in this unit by Mr Paul Webb using the method of Adams *et al.* (1984). MMC was obtained from Sigma Chemicals and dissolved in phosphate-buffered saline immediately before use, to give 2 mg $ml^{-1}$ stock solution. SR4233 was a gift from Drs J. Martin Brown and Ven Narayanan (Department of Therapeutic Radiology, Stanford University, USA and the National Cancer Institute respectively).

### Cell culture

MMC mutant cell lines and CHO-K1 cells were maintained in monolayer cultures using HAM's F12 culture medium (Gibco) supplemented with 2 mM L-glutamine, penicillin, streptomycin, 5% new born calf serum and 5% fetal calf serum (Biological Industries). V79 and *irs* cells were grown in monolayer cultures using MEM (Gibco) medium supplemented with glutamine, penicillin, streptomycin and 10% fetal calf serum (Biological Industries). Cultures were incubated at 37°C in a humidified atmosphere of 5% $CO_2$ in air, and passaged twice weekly.

### Drug treatments

Exponentially growing cells were harvested from monolayer cultures, diluted and appropriate cell numbers plated out in medium, on glass or plastic Petri dishes for hypoxic or aerobic drug exposures. Cells were allowed to attach for 2 h before drug treatment. All drugs were diluted in tissue culture medium and added to cells for 3 h. For aerobic treatments cells were incubated at 37°C for 3 h in an humidified atmosphere of 5% $CO_2$ in air. For hypoxic exposure drug was added to the cells on glass Petri dishes and immediately gassed with $N_2$ + 5% $CO_2$ at 37°C for 3 h. The drug-containing medium was then removed from the dishes, replaced with fresh medium and cells assayed 7-10 days later for colony formation.

### Results

### *MMC cells*

These cell lines have been isolated on the basis of their sensitivity or resistance to mitomycin C following aerobic exposure to the drug. Each mutant cell line and the wild type CHO-K1 cells were treated with MMC, RSU-1069 and SR4233 under both aerobic and hypoxic conditions to assess their cross-sensitivities to other bioreductive drugs.

*Mitomycin C* Figure 1 shows the survival curves from CHO-K1 and the MMC cell lines MMC-2 and MMC-3 following 3 h aerobic or hypoxic exposure to MMC. In each of the three cell lines the drug was slightly more toxic in hypoxic cells compared to the effect in aerobic cells but the differential toxicities were small: 1.1 for CHO-K1, 1.5 for MMC-2 and 1.2 for MMC-3 cell lines. (Differential toxicity is defined as the ratio of the concentrations of drug required to reduce cell survival to 10% under aerobic and hypoxic conditions.) Under aerobic conditions $MMC^r$ cells were 20 times more resistant to MMC than the parent cell line but only two-fold resistant under hypoxic conditions (Table I). These data suggest that the $MMC^r$ cells differ in the way they metabolise MMC compared to the other cell lines. Furthermore the difference in hypoxic sensitivity of the wild type and $MMC^r$ cells is small compared to the difference observed in air which suggests that differences between the two cell lines may lie in the ability to activate MMC under aerobic conditions. The observation that both MMC-2 and MMC-3 cell lines are more sensitive to MMC compared to CHO-K1, under both aerobic and hypoxic conditions suggests that the oxygenation status is not important for the enhanced sensitivity.

**Table I** Differential toxicities and drug concentrations required to reduce CHO-K1 and MMC-cell survival to 10% following exposure to bioreductive drugs

| *Cell line* | *Drug* | *Aerobic exposure* | *Hypoxic exposure* | *Differential toxicity* |
|---|---|---|---|---|
| CHO-K1 | MMC (μM) | 2.9 | 2.7 | 1.1 |
| MMC-2 | | 2.3 | 1.5 | 1.5 |
| MMC-3 | | 1.2 | 1.0 | 1.2 |
| $MMC^r$ | | 53 | 5.3 | 10.0 |
| CHO-K1 | RSU-1069 (mM) | 0.49 | 0.098 | 5.0 |
| MMC-2 | | 0.20 | 0.037 | 5.4 |
| MMC-3 | | 0.47 | 0.11 | 4.3 |
| $MMC^r$ | | 0.55 | 0.12 | 4.5 |
| CHO-K1 | SR4233 (mM) | 0.33 | 0.012 | 28 |
| MMC-2 | | 0.28 | 0.013 | 22 |
| MMC-3 | | 0.25 | 0.017 | 15 |
| $MMC^r$ | | 0.50 | 0.024 | 21 |

*RSU-1069* The cross-sensitivity of MMC cells to RSU-1069 was also investigated. Survival curves revealed a pattern

**Figure 1** Survival curves for CHO-K1 and MMC cells following 3 h exposure to MMC under aerobic (○) or hypoxic (●) conditions. Points represent data pooled from three experiments.

different to that observed with MMC. The sensitivities of MMC-3 and $MMC^r$ cells to RSU-1069 were similar to that of CHO-K1 wild type under both aerobic and hypoxic conditions. However, MMC-2 cells were 2–3 times more sensitive than CHO-K1 cells in air and nitrogen. Differential toxicities of about 5 (Table I) were observed for CHO-K1 and mitomycin C sensitive or resistant cells alike. Since the $MMC^r$ cells were not resistant to RSU-1069 under aerobic conditions, it seems likely that this class of compound is activated into a cytotoxic state by a different pathway to that of MMC.

*SR4233* Each cell line was considerably more sensitive to SR4233 under hypoxic conditions than in oxygen. The differential toxicities were between 15 (MMC-3) and 28 (CHO-K1) (Table I). The MMC-sensitive cell lines (MMC-2 and MMC-3) both showed sensitivities similar to that of CHO-K1 cells under both aerobic and hypoxic conditions (Table I). However, it is notable that $MMC^r$ cells were twice as resistant as the CHO-K1 cells to SR4233 under both exposure conditions. Results from the experiments in which MMC cells were treated with other classes of bioreductive compound suggest that each drug is metabolised differently. However, a slight aerobic cross-resistance ($<2$) has been observed in $MMC^r$ cells following exposure to SR4233. This was not as large as that which has been observed following treatment with MMC but may indicate that there is a step common to the metabolism of both drugs in these cells.

*irs cells*

In a parallel study three cell lines sensitive to ionising radiation and the V79 wild type were also treated with MMC, RSU-1069 and SR4233. *irs 1* and *irs 2* are about three times more sensitive and *irs 3* twice as sensitive to X-rays compared to V79 cells (Jones *et al.*, 1987). These cell lines also have variable sensitivities to other DNA damaging agents, for example UV irradiation, EMS and mitomycin C (Jones *et al.*, 1987). Jones *et al.* (1987) have shown that *irs 1* cells were 60-fold more sensitive to MMC following a 2 h exposure under aerobic conditions compared to the parent cell line, and also showed the greatest sensitivities to UV and EMS. *irs 2* and *irs 3* were more sensitive than V79 to each agent but the differences were small compared to those observed for *irs 1*.

*Mitomycin C* In the present study *irs 1* was found to be at least 25 times more sensitive to MMC (following a 3 h exposure under aerobic conditions) than V79 cells (Table II). *irs 2* cells were less sensitive and *irs 3* equally as sensitive as the parent cells in air, but MMC was more toxic to all cell lines exposed under anaerobic conditions. However, apart from *irs 2* the differential toxicities observed were small (Table II). *irs 2* cells have a differential toxicity of 8, four times greater than that of V79 cells. This increased differential is mainly due to the relative resistance of these cells to MMC under aerobic conditions, compared with the sensitivities of the other cell lines. *irs 2* are also slightly more sensitive than V79 cells following hypoxic exposure. There does not appear to be any correlation between X-ray sensitivity and sensitivity to MMC for the *irs* cell lines.

**Table II** Differential toxicities and drug concentrations required to reduce V79 and *irs* cell survival to 10% following exposure to bioreductive drugs

| Cell line | Drug | *Aerobic exposure* | *Hypoxic exposure* | *Differential toxicity* |
|---|---|---|---|---|
| V79 | MMC (μM) | 2.1 | 1.0 | 2.1 |
| *irs 1* | | 0.085 | 0.068 | 1.2 |
| *irs 2* | | 5.8 | 0.73 | 8.0 |
| *irs 3* | | 2.2 | 0.85 | 2.5 |
| V79 | RSU-1069 (mM) | 0.75 | 0.015 | 50 |
| *irs 1* | | 0.075 | 0.0075 | 10 |
| *irs 2* | | 0.39 | 0.014 | 27 |
| *irs 3* | | 0.20 | 0.01 | 20 |
| V79 | SR4233 (mM) | 0.30 | 0.005 | 60 |
| *irs 1* | | 0.02 | 0.0028 | 7.2 |
| *irs 2* | | 0.065 | 0.0027 | 23.0 |
| *irs 3* | | 0.05 | 0.0026 | 19 |

*RSU-1069* A different pattern of cell survival has been observed following treatment of *irs* cells with RSU-1069. Figure 2 shows that RSU-1069 is preferentially toxic to hypoxic cells in all four cell lines. V79 cells show a differential toxicity of 50 but this value is reduced to 26 and 20 for *irs 2* and *irs 3* respectively. *irs 1* cells have a differential toxicity of only 10 (Table II). This is because *irs 1* are 10 times more sensitive than V79 under aerobic conditions although both lines show similar hypoxic response.

Comparison of the survival curves for *irs* and V79 cells treated wtih RSU-1069 under either aerobic or hypoxic conditions, reveals that all lines show similar sensitivities to the drug under hypoxic conditions. However, under aerobic conditions *irs* cells were between 2 and 10 times as sensitive to RSU-1069 as V79 wild type cells.

*SR4233* Figure 3 compares the aerobic and hypoxic survival curves of *irs* cells treated with SR4233. A differential toxicity of 60 calculated for V79 cells is reduced to between 23 and 7.2 for the radiation sensitive cell lines (Table II). All cell lines were more sensitive when exposed to SR4233 in anoxia but as was observed for RSU-1069 (Figure 2), the hypoxic survival curves of *irs* cells were very similar to that observed for V79 cells. The reduction in differential toxicity is again due therefore, to differences in the aerobic toxicities of the *irs* compared to V79 cells, where *irs* cells were up to 15 times more sensitive.

The results described above show that there is little difference in the sensitivity of the mutant and wild type cells to either RSU-1069 or SR4233 under hypoxic conditions. Furthermore these data suggest that the *irs* cells have not lost their capacity to reduce and activate the compounds under hypoxic conditions.

### Discussion

The ultimate biological effects of DNA damage induced either chemically or by radiation depend on a variety of factors of which potential for repair may be the most significant. A comparison of the response to DNA damage in cells selected for their sensitivity to different genotoxic agents should give some insight not only to the nature of the sensitivity but also the mode of action by which these genotoxic agents kill cells.

The CHO cells were selected for this study on the basis of their different sensitivities to MMC in air. These cells do not show any patterns of cross-resistance or sensitivity towards RSU-1069 or SR4233 in air, although MMC-2 cells show a small increase in sensitivity to RSU-1069 and $MMC^r$ cells are slightly more resistant to SR4233 than the parent cell lines.

Under aerobic conditions the $MMC^r$ cells are at least 20 fold resistant to MMC compared to the CHO-K1 cells, but this resistance is almost completely overcome under hypoxic conditions (Hoban *et al.*, 1990). This results in a differential toxicity of 10 for this cell line following exposure to MMC. In contrast, there is only a slight increase in the toxicity of MMC in the other cell lines in nitrogen. This suggests that the CHO cell lines with the exception of $MMC^r$ cells can activate MMC almost equally well under aerobic and hypoxic conditions. It is only under conditions of low oxygenation that $MMC^r$ cells have the ability to reduce and thereby activate MMC.

Support for this proposal comes from the recent studies of Hoban *et al.* (1990) on the levels of various enzymes in CHO cells that are capable of activating MMC. Both the CHO-K1 and $MMC^r$ cells lack measurable DT-diaphorase activity but, in particular, the $MMC^r$ cells show a 3–4-fold lower level of

**Figure 2** Survival curves for V79 and *irs* cells following 3 h exposure to RSU-1069 under aerobic (○) or hypoxic (●) conditions. Each point represents the mean of data from three experiments and error bars (s.e.m.) are included where they exceed the size of the symbol used.

**Figure 3** Survival curves for V79 and *irs* cells following 3 h exposure to SR4233 under aerobic (open symbols) or hypoxic (closed symbols) conditions. ○●, V79; △▲, *irs 1*; □■, *irs 2*; ◇◆, *irs 3*.

NADPH cytochrome P450 reductase compared to the CHO-K1 cells. Both these enzymes have been implicated in the bio-activation of MMC (Keyes *et al.*, 1984; Schlager & Powis, 1988; Marshall *et al.*, 1989; Dulhanty *et al.*, 1989) and it is this P450 enzyme that is considered to be important for activation of MMC in the CHO cells (Hoban *et al.*, 1990). Thus, the lower level of P450 reductase in the $MMC^r$ cells may be insufficient to allow activation of the drug in air but not under hypoxic conditions.

The values of differential toxicity for RSU-1069 are about 5 for each of the CHO cell lines. It is known that NADPH cytochrome P450 reductase plays an important part in the reductive activation of the 2-nitromidazole, benznidazole (Walton *et al.*, 1989). Presumably this enzyme would also be important for activation of RSU-1069. However, the differences in enzyme levels between the $MMC^r$ and CHO-K1 cells do not lead to significant differences in toxicity for this drug. Similarly, there is little difference in the absolute toxicity of SR4233 in either air or $N_2$ for these cell lines, although this drug is known to be metabolised primarily by cytochrome P450 in microsomal preparations (Walton *et al.*, 1989).

The *irs* cells are known to belong to different genetic complementation groups. Therefore the radiosensitivity of each cell line may reflect a mutation, inactivation or deletion of a different gene(s) in each case, although they may be related. These lines are known to express a range of sensitivities to other genotoxic agents (Jones *et al.*, 1987). The present results show that they also exhibit variable sensitivities to three classes of bioreductive compound. Both V79 cells and radiation-sensitive cell lines are more sensitive to all three drugs under hypoxic conditions compared to air. *irs 1* cells are much more sensitive to MMC under both aerobic and hypoxic conditions compared to V79 cells. In contrast, we have found that *irs 2* cells show MMC toxicity equivalent to that of V79 cells under nitrogen but appear to be 2-fold resistant in air. As discussed for the MMC$^r$ cells, this may indicate that *irs 2* cells are either unable to activate MMC adequately in air, or may possess a repair mechanism capable of repairing or protecting against the damage produced by MMC in the presence of oxygen.

Treatment of *irs* cell lines with RSU-1069 and SR4233 has shown that each of the mutant cell lines is considerably more susceptible to the aerobically induced drug damage than are V79 cells. Each line (V79 and *irs*) are equally sensitive to these two drugs under anoxia compared to the range of sensitivities shown in air. For each drug, the mechanism of cytotoxic action is different in aerobic cells compared with that in hypoxic cells. For example, RSU-1069 is known to act as a monofunctional agent in air but is converted to a bifunctional reacting species when the compound is reduced under poorly oxygenated conditions (Stratford *et al.*, 1986). The different aerobic responses of these cell lines to the drugs suggest that in air they have a reduced capacity to cope with the drug-induced damage compared to that of V79 cells. One possibility is that the *irs* cells lack a DNA repair facility normally present in V79 cells such that doses which are not toxic in wild type V79 cells become lethal in the mutant cell lines. It has been demonstrated, however (Thacker & Ganesh, 1990), that none of the *irs* cell lines are deficient in the mechanism necessary for the rejoining of single or double strand DNA breaks following γ-irradiation.

The *irs* cells are radiation sensitive and it is known that a substantial proportion of damage caused by radiation is oxidative, i.e. involving OH radicals. The superoxide radical, $O_2^-$, is formed as a consequence of futile one-electron reduction of bioreductive drugs in the presence of oxygen (Mason & Holtzman, 1975; Wardman & Clarke, 1976; Kirkpatrick, 1989). The radical can, under appropriate conditions, lead to the formation of the ·OH radical. Therefore cellular sensitivity to oxidative damage could provide an explanation for the sensitivity of the *irs* cells to RSU-1069 and SR4233 in air as well as their radiation sensitivity. This proposal is currently being evaluated.

Mechanisms involving drug transport, drug activation, drug detoxification and the elimination of, or repair of, drug induced lesions may each influence the levels of drug sensitivity observed in the mutant cell lines. The present results show that under hypoxia differences in the efficiency of any of these processes are unimportant in *irs* cells since the cytotoxicities are fairly constant. This appears not to be so for aerobic cells. Further studies are necessary in order to identify which of these processes are affected in the mutant cell lines. It is clear however that the variability in the radiation sensitivities of these lines is not related to their abilities to metabolically reduce, or activate the drugs under hypoxic conditions.

The authors would like to thank Miriam Stephens for her technical assistance, Paul Webb for the synthesis of RSU-1069 and Mrs Anne Johnson for the typing of this manuscript. Thanks are also due to Dr J. Thacker for the *irs* cells and Dr I. Hickson for the MMC cells. This work was supported by NCI Grant No. RO1 CA44 126-01.

### References

ADAMS, G.E.A., AHMED, I., SHELDON, P.W. & STRATFORD, I.J. (1984). RSU-1069, a 2-nitroimidazole containing an alkylating group: high efficiency as a radio- and chemosensitizer *in vitro* and *in vivo*. *Int. J. Radiat. Oncol. Biol. Phys.*, **10**, 1653.

DULHANTY, A.M., LI, M. & WHITMORE, G.F. (1989). Isolation of Chinese hamster ovary mutants deficient in excision repair and mitomycin C bioactivation. *Cancer Res.*, **49**, 117.

HOBAN, D.J., WALTON, M.I., ROBSON, C.N. & 5 others (1990). Mitomycin C resistance under aerobic but not hypoxic conditions in a mammalian cell line: association with impaired drug activation and decreased NADPH cytochrome P450 reductase activity. *Cancer Res.* (in the press).

JONES, N.J., COX, R. & THACKER, J. (1987). Isolation and cross-sensitivity of X-ray-sensitive mutants of V79-4 hamster cells. *Mutation Res.*, **183**, 279.

KENNEDY, K.A., TEICHER, B.A., ROCKWELL, S. & SARTORELLI, A.C. (1980). The hypoxic tumour cell: a target for selective cancer chemotherapy. *Biochem. Pharmacol.*, **29**, 1.

KEYES, S.R., FRACASSO, P.M., HEIMBROOK, D.C., ROCKWELL, S., SLIGAR, S.G. & SARTORELLI, A.C. (1984). Role of NADPH-cytochrome C reductase and DT-diaphorase in the biotransformation of mitomycin C. *Cancer Res.*, **44**, 5638.

KIRKPATRICK, D.L. (1989). The development of hypoxic tumor cell cytotoxic agents. *Pharmacol. Ther.*, **40**, 383.

MARSHALL, R.S., PATERSON, M.C. & RAUTH, A.M. (1989). Deficient activation by a human cell strain leads to mitomycin resistance under aerobic but not hypoxic conditions. *Br. J. Cancer*, **59**, 341.

MASON, R.P. & HOLTZMAN, J.L. (1975). The role of catalytic superoxide formation in the $O_2$ inhibition nitroreductase. *Biochem. Biophys. Res. Commun.*, **67**, 1267.

ROBSON, C.N., HARRIS, A.L. & HICKSON, I.D. (1985). Isolation and characterisation of Chinese hamster ovary cell lines sensitive to Mitomycin C and Bleomycin. *Cancer Res.*, **45**, 5304.

SCHLAGER, J.J. & POWIS, G. (1988). Mitomycin C is not metabolised by, but is an inhibitor of human kidney NAD(P)H: (quinone-acceptor) oxoreductase. *Cancer Chemother. Pharmacol.*, **22**, 126.

STRATFORD, I.J., O'NEILL, P., SHELDON, P.W., SILVER, A.R.J., WALLING, J.M. & ADAMS, G.E.A. (1986). RSU-1069, A nitroimidazole containing an aziridine group. Bioreduction greatly increases cytotoxicity under hypoxic conditions. *Biochem. Pharmacol.*, **35**, 105.

THACKER, J. & GANESH, A.N. (1990). DNA-break repair, radioresistance of DNA synthesis and campothecin sensitivity in the radiation-sensitive *irs* mutants: comparison to ataxia-telangectasia cells. *Mutation Res.* (in the press).

WALTON, M.I., WOLF, R.C. & WORKMAN, P. (1989). Molecular enzymology of the reductive bioactivation of hypoxic cell cytotoxins. *Int. J. Radiat. Oncol. Biol. Phys.*, **16**, 983.

WARDMAN, P. & CLARKE, E.D. (1976). Oxygen inhibition of nitroreductase: electron transfer from nitro radical-anions to oxygen. *Biochem. Biophys. Res. Commun.*, **69**, 942.

ZEMAN, E.M., BROWN, J.M., LEMMON, M.J., HIRST, V.K. & LEE, W. (1985). SR4233: a new bioreductive agent with high selective toxicity for hypoxic mammalian cells. *Int. J. Radiat. Oncol. Biol. Phys.*, **12**, 1239.

ZEMAN, E.M., BAKER, M.A., LEMMON, M.J. & 5 others (1989). Structure-activity relationships for benzotriazine di-*N*-oxides. *Int. J. Radiat. Oncol. Biol. Phys.*, **16**, 977.